# Electrochemically-mediated selective capture of heavy metal chromium and arsenic oxyanions from water

**DOI:** 10.1038/s41467-018-07159-0

**Published:** 2018-11-08

**Authors:** Xiao Su, Akihiro Kushima, Cameron Halliday, Jian Zhou, Ju Li, T. Alan Hatton

**Affiliations:** 1 Department of Chemical Engineering, MIT, 77 Massachusetts Avenue, Cambridge, MA 02139 USA; 2 Department of Nuclear Engineering, MIT, 77 Massachusetts Avenue, Cambridge, MA 02139 USA; 3 Present Address: Department of Materials Science and Engineering, University of Central Florida, 12760 Pegasus Drive, Orlando, FL 32816 USA

## Abstract

The removal of highly toxic, ultra-dilute contaminants of concern has been a primary challenge for clean water technologies. Chromium and arsenic are among the most prevalent heavy metal pollutants in urban and agricultural waters, with current separation processes having severe limitations due to lack of molecular selectivity. Here, we report redox-active metallopolymer electrodes for the selective electrochemical removal of chromium and arsenic. An uptake greater than 100 mg Cr/g adsorbent can be achieved electrochemically, with a 99% reversible working capacity, with the bound chromium ions released in the less harmful trivalent form. Furthermore, we study the metallopolymer response during electrochemical modulation by in situ transmission electron microscopy. The underlying mechanisms for molecular selectivity are investigated through electronic structure calculations, indicating a strong charge transfer to the heavy metal oxyanions. Finally, chromium and arsenic are remediated efficiently at concentrations as low as 100 ppb, in the presence of over 200-fold excess competing salts.

## Abstract (web summary)

Chromium and arsenic are prevalent water pollutants, but their removal is currently limited by low selectivity. Here, the authors use redox-active metallopolymer electrodes based on poly(vinyl)ferrocene to selectively remove the two heavy metal oxyanions at concentrations as low as 100 ppb.

## Introduction

The issues of anthropogenic water pollution and geographical scarcity of clean water on a global scale are some of the main engineering challenges of the twenty-first century^1^. Heavy metal contaminants have been a major health and environmental hazard across the world. Chromium, in particular, is a priority target for the Environmental Protection Agency (EPA) due to its prevalence and high degree of toxicity^2–4;^ it is among the 10 most frequently detected ground water contaminants at hazardous waste sites, and one of the 14 most toxic heavy metals, especially when present in its hexavalent, oxyanion state (e.g., Cr_2_O_7_^2−^, CrO_4_^2−^, HCrO_4_^−^)^5,6^. Major sources of pollution include tanneries and metal plating, and are of concern both in the United States and the developing world. Whereas trivalent Cr(III) is a naturally occurring form in the earth’s crust, hexavalent Cr(VI) is an anthropogenic chemical^7^—as such, the development of energy-efficient pathways for capture of Cr(VI) and conversion to Cr(III) is key to long-term environmental sustainability^2,6^. Arsenic is another heavy metal in its highly soluble oxyanion form that has received strong interest both in North America and developing countries due to its acute health effects^8,9^, and wide prevalence from both natural and anthropogenic sources^10^. Conventional methods for heavy metal removal involve ion exchange or chemical resins for adsorption, which often require co-reagents or excessive regeneration chemicals^11^, and can suffer from slow kinetics with processing times on the order of hours^12–14^.

The development of smart materials for water purification and environmental remediation has received intense attention recently, with micropollutants and trace contaminants being key areas of concern^15^. Electrosorption-based processes often offer an attractive platform due to their modularity and the absence of a chemical regeneration step, based on a wide variety of material platforms for both deionization^16–19^ and selective ion removal^20–22^. Redox-active materials have recently been demonstrated to be a promising platform for separations due to their molecular selectivity and electronic tunability^23,24^. Metallopolymer-based electrodes, especially poly(vinyl)ferrocene (PVF), have shown remarkable ion-uptake capacities for organic contaminants based on electrochemically activated chemical interactions^23–25^. The main features of these systems are the fast electron transfer and redox processes at moderate potentials (<1.2 V vs. standard hydrogen electrode (SHE)), which allow reversible adsorption and desorption of ionic components by electrochemical modulation of charge and hydrogen-bonding interactions between carboxylates and the metallopolymers. A universal redox material platform, targeting the molecular level recognition of heavy metal oxyanions (HMOAs), would overcome a major challenge for environmental remediation, especially in the presence of competing excess anions. Furthermore, the nature of the interaction of these metallopolymers with relevant transition metals has not yet been fully explored.

In the current work, PVF-functionalized electrodes are applied in the removal of anionic chromium and arsenic oxyanions, under a range of different concentrations and electrolyte conditions. In addition, we provide insight into the transformation of chromium, which is reduced to its trivalent form during release on reduction of the metallopolymer adsorbent. From a general perspective, we demonstrate the high selectivity of Faradaic electrodes for HMOA contaminants, with the adsorption and desorption controlled purely by electrochemical modulation. The fundamental interactions regulating the selectivity are shown to depend strongly on solvation effects and on the charge transfer characteristics of the ion pair, regulated by the electronic structure of the anions and overcoming simple electrostatic attractions. In addition, we pursue the nanoscale observation of our metallopolymer film under ion adsorption using in situ electrochemical transmission electron microscopy (TEM). In situ TEM allows the direct high-resolution imaging and observation of an electrochemical response of a material interface, and can thus shed light on the kinetics and morphological changes during ion insertion in a direct manner. Our results are expected to serve as an important platform for tailoring advanced redox materials targeting heavy metal recovery and remediation.

## Results

### Electrosorption of chromium

**Fig. 1:**
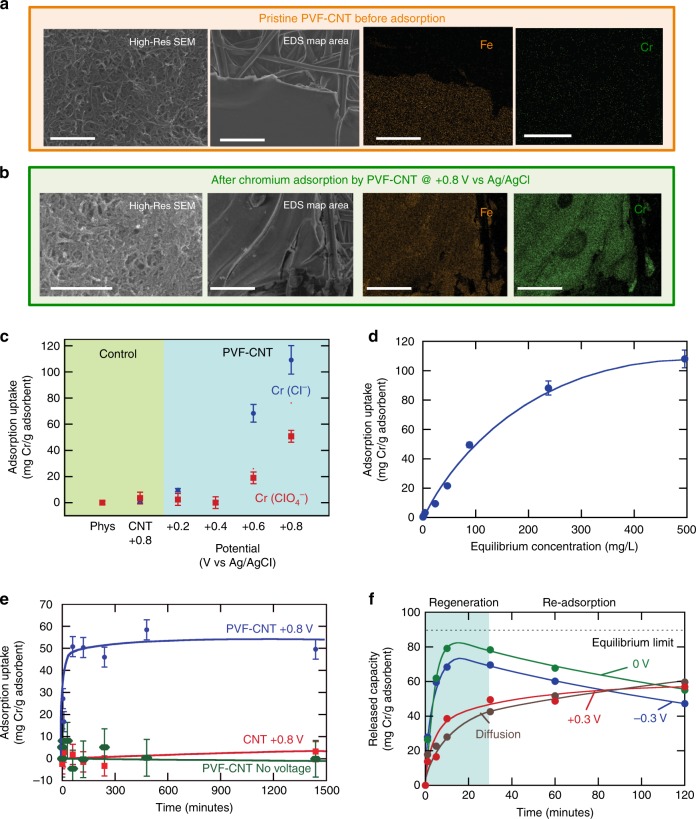
Electrosorption of chromium oxyanions by PVF-CNT. **a** Scanning electron microscopy (SEM) images of the PVF-CNT electrodes before the adsorption of chromium, and EDS mapping of the iron of the metallopolymer, and of adsorbed Cr. **b** SEM micrographs and EDS mapping after chromium adsorption. The area corresponding to the PVF is also saturated with the adsorbed Cr. **c** Comparison of chromium-uptake capacity of PVF-CNT and CNT electrodes, for 1 mM (NH)_4_Cr_2_O_7_ in 20 mM NaClO_4_ and in 20 mM NaCl. No Cr adsorption occurs on a PVF-CNT electrode without potential, and a much higher adsorption capacity for PVF-CNT than pristine CNT is observed at +0.8 V vs. Ag/AgCl potential adsorption. **d** Equilibrium isotherm for PVF-CNT at +0.8 V vs. Ag/AgCl adsorption for a range of (NH)_4_Cr_2_O_7_ concentrations in 20 mM NaClO_4_. **e** Adsorption kinetics for Cr at 1 mM (NH)_4_Cr_2_O_7_ in 20 mM NaClO_4_. **f** Effect of discharge potential on regeneration efficiency of the PVF-CNT electrodes, measured by the normalized mass of Cr released (mg Cr/g PVF polymer). Electrodes were charged at +0.8 V (vs. Ag/AgCl) and discharged at 0, −0.3, and +0.3 V into 20 mM NaClO_4_, and dynamic results were compared to a diffusion-controlled case in which the electrode was left in the desorption solution under no applied potential. Scale bar for high-resolution SEM is 800 nm, and scale bar for EDS and Fe/Cr mapping is 50 μm. Error bars represent standard deviations from three replicate measurements

The effectiveness of PVF as a heterogeneous, electrochemically mediated adsorbent for the removal of chromate from water was evaluated using uniform, nanostructured films of multiwalled carbon nanotubes (CNTs) and PVF prepared by a drop-casting method^23,26^. The nanoporous structure of the film was observed clearly by high-resolution scanning electron microscope (Fig. 1a), with the iron content confirmed by energy-dispersive spectroscopy (EDS). Chromium adsorption was carried out from a mixture of 1 mM (NH_4_)_2_Cr_2_O_7_ and 20 mM ClO_4_^−^ under chronoamperometric conditions at +0.8 V vs. Ag/AgCl (starting current ~2 mA/cm^2^ as seen in Supplementary Figure 8). After 2 h of adsorption, the increase in chromium on the PVF-CNT film was evident through EDS mapping (Fig. 1b). X-ray photoelectron spectroscopy (XPS) surface analysis also confirmed the high uptake of chromium relative to the CNT control, in which no visible chromium adsorption was observed (Supplementary Figures 13 and 14). At 10 mM Cr_2_O_7_^2−^ and 20 mM ClO_4_^−^ (equimolar chromium and perchlorate concentrations in the mixture), a molar comparison found a close to stoichiometric ratio between chromium and iron, indicating the strong chromate-ferrocenium binding as an ion pair (Fe2p 1.25:1 Cr2p ratio, Cl <0.1%, from high-resolution XPS quantitation). With chromate as the minority ion (1 mM Cr_2_O_7_^2−^ and 20 mM ClO_4_^−^), the degree of competing perchlorate adsorption increased (Cr2p 1.3:1 Cl2p ratio), with the iron to chromium ratio approaching 4:1 (from both survey and high-resolution scans), which approximately correlates with the experimental adsorption capacity of ~60 mg/g (Fig. 1c). A total elemental analysis from the survey scans also points to a certain degree of cation (Na^+^ and NH_4_^+^) insertion within the film (Supplementary Figure 13) due to charge compensation to ensure electroneutrality.

On varying the potential applied to the PVF-CNT electrode, we noticed that chromium adsorption was the highest under full oxidation of PVF (at +0.8 V vs. Ag/AgCl), with little to no adsorption below +0.4 V (Fig. 1c), with either perchlorate or chloride as the supporting electrolyte. Under higher potentials, more ferrocene (Fc) units in the electrode become oxidized to allow for chromate binding. As will be discussed later, these observations are also in agreement with the results from in situ TEM, which showed polymer expansion at the peak redox potential of the cyclic voltammetry (CV) (see Supplementary Movies 1–5). An equilibrium isotherm at +0.8 V vs. Ag/AgCl shows that a maximum capacity of up to 100 mg chromium/g adsorbent can be achieved in perchlorate (Fig. 1d), which is higher than loadings reported for various ion-exchange materials and nanoparticle sorbents^14,27;^ the advantage of our redox electrodes is that they are fully electrochemically controlled and do not require the addition of chemical regenerants to release the captured ions, with remarkably higher selectivity and adsorption uptake than conventional carbon electrodes^28^. In addition, the adsorption reaches fast equilibrium across a range of concentrations (Fig. 1e and Supplementary Figure 10), which is remarkable in comparison to various ion-exchange or other electrosorption systems, which often require hours to reach adsorption equilibrium^12–14^. These fast adsorption rates are comparable to capacitive methods in deionization^16–19^, with the added advantage of the high selectivity towards the select chromium anions in competition with interfering chloride ions. Neither a control CNT electrode (pristine CNT at +0.8 V vs. Ag/AgCl) nor the neutral PVF-CNT (no voltage applied) exhibited affinity for the dichromate over the competing electrolyte (Fig. 1c, e). Finally, it must be noted that comparable chromium uptakes can be obtained by applying chronopotentiometric charging (at current densities varying from 0.1 to 1 mA/cm^2^) and also with capacitive counter electrodes made of porous carbons (see Supplementary Figure 11).

### Regeneration and spectroscopic analysis

**Fig. 2:**
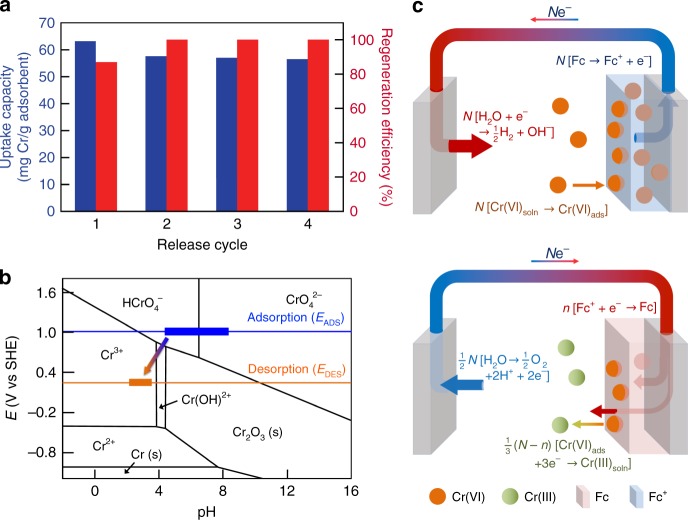
Investigation of the reversibility of the electrochemically mediated capture-and-release process. **a** Recyclability of the electrode over a number of discharge cycles (+0.8 V adsorption, 0 V discharge), as given by the normalized mass released (in blue), and the regeneration efficiency (%) (in red), the latter denoting the relative amount of Cr recovered relative to that adsorbed in each cycle. **b** The *E*-pH diagram for chromium speciation predominance, constructed with commercial thermochemical software (FactSage) at 25 °C for 1 mM total chromium concentration in the liquid phase. The adsorption and desorption potentials are noted, with the range of solution conditions marked both for adsorption (in blue) and desorption (in orange). **c** The Faradaic reactions occurring at the surface of the electrode pair are shown during both adsorption and release. During adsorption, hexavalent chromate is captured by the anode through selective binding, whereas during release, reduction of ferrocenium to ferrocene and of Cr(VI) to Cr(III) occurs

Regeneration of the redox electrodes was accomplished through application of a lower potential to reduce Fc^+^ and release the adsorbed chromium. A series of release conditions were tested including −0.3, 0, and +0.3 V and no applied potential (results shown in Fig. 1f). The release of up to 87% of the adsorbed chromium was achieved at 0 V vs. Ag/AgCl. A cycling study was carried out with the electrode charged at +0.8 V vs. Ag/AgCl, in the presence of 1 mM chromate for 10 min, and discharged at 0 V vs. Ag/AgCl into a clean electrolyte solution for 30 min, which was then assayed for released chromium. Figure 2a shows that the working capacity of the electrode was retained at close to 55 mg Cr/g adsorbent for a number of cycles with 100% regeneration efficiency per step starting at cycle 2. In addition, no metallopolymer leaching was observed in a inductively coupled plasma (ICP) assay for Fe in the liquid phase, or from an XPS analysis of the PVF surface (equivalent iron % before and after).

An illustrative scheme showing the full adsorption and release cycle is shown in Fig. 2c. A closer look at the voltage on the *E*-pH (aka Pourbaix) diagram can shed light on the pathways for chromium transformation during this electrochemical process (Fig. 2b). The pH of the adsorption mixture was initially ~pH = 5, and slowly increased during adsorption due to both water and oxygen reduction at the counter electrode (Supplementary Figure 16, Supplementary Figure 17, and Supplementary Figure 18). During discharge, the pH sharply dropped to ~3.2–3.5 due to water oxidation at the counter electrode. During the adsorption step, an electrochemical potential above +0.8 V vs. Ag/AgCl, or 1.0 V vs. SHE, was applied, a range over which the anionic chromium dominates (CrO_4_^2−^, HCrO_4_^−^, or Cr_2_O_7_^2−^, depending on the pH and total chromium concentration). Over the range of concentrations tested (0.1–10 mM) and at pH = 5, HCrO_4_^−^ is the predominant species prior to adsorption. As shown by XPS, in situ TEM, and the various adsorption tests, there is a strong selectivity of PVF for the chromate species over other competing anions during electrosorption following oxidation of the PVF-CNT electrode. To promote discharge of the captured ions, a lower reduction potential was applied (0 V vs. Ag/AgCl, +0.2 V vs. SHE), which brought the system down to a region in which cationic forms of chromium predominate (e.g., Cr(OH)_3 − *x*_^*x*+^). A negative potential (−0.3 V vs. Ag/AgCl), even though it promoted a rapid desorption of the chromium from the Fc film, resulted in chromium re-adsorption onto the negatively polarized electrode after a certain period of time, probably due to the cationic predominance of Cr^3+^ and Cr(OH)_2_^+^. On the other hand, the +0.3 V run showed a discharge profile comparable to that of the diffusion-controlled release of chromium (Fig. 1f), pointing to a slower reduction of the Fc units and subsequent release of chromium. The higher release at an applied potential of 0 V implies an optimal tradeoff between reduction of the metallopolymer and re-adsorption of any cationic chromium.

The spontaneous conversion of Cr(VI) to Cr(III) at the surface of the electrodes was corroborated by the XPS observation of a significant accumulation of Cr(OH)_3 − *x*_^*x*+^ on the electrode, based on the binding energy shift (Cr 2p_1/2_ at 587.2 eV and Cr 2p_3/2_ at 577.7 eV)^29^, as well as the Cr(VI) shoulder at 580 eV (Supplementary Figure 14). The XPS profile supports the supposition of a surface-induced conversion of Cr(VI) to Cr(III)^29^. Chromate reduction by free ferrous iron (Fe(II)), in which water acts as a co-reagent, has been reported for natural aquatic systems^30^. Our method couples an electrochemical reduction step following removal of the chromate ions from the feed solution during the oxidation step. A liquid-phase spectrophotometric assay to evaluate the degree of Cr(VI) conversion to Cr(III) in the aqueous phase before and after reduction showed that both the stock solution (containing 1 mM NH_4_Cr_2_O_7_) and the supernatant after adsorption contained Cr(VI) as the majority species (>98%). The preservation of the hexavalent form of chromium even after electrochemical treatment under oxidation indicated that application of +0.8 V vs. Ag/AgCl does not change the oxidation state of bound Cr(VI). Also, the neutral Fc polymer showed no molecular affinity for the anions, as both XPS and spectrophometric assays indicated no conversion of Cr(VI) to Cr(III). However, after discharge at 0 V vs. Ag/AgCl, 81% of the released chromium was found to be in the trivalent state of Cr(III), providing strong evidence that the electrochemically mediated release step promotes the transformation.

Here, we must note the importance of the anode in enabling the synergistic reduction of the chromium oxyanion at the cathode during the release process (Fig. 2c). Not only does water oxidation at the anode provide the electrons for reduction of both PVF^+^ and Cr(VI), but the protons produced are crucial in that they regulate the pH towards a range in which the cationic species are soluble (see the *E*-pH diagram in Fig. 2b), and, conveniently, act as co-reagents in the reduction of Cr(VI) to Cr(III) species^30,31^. A charge balance on the electrochemical system for electrons transferred to the cathode for Cr(VI) and PVF^+^ reduction, and from the anode, with the production of protons, gives a cathode current efficiency of around 100%; the calculated value of ~1.27 Coulomb transferred to the cathode, based on the assumption of full Fc reduction coupled with the equivalent Cr(VI) transformation, agreed well with the experimentally determined value of 1.24 Coulomb of charge transferred during regeneration (Supplementary Figure 9). The balance of protons (based on pH change and estimated consumption during dichromate conversion) gives a theoretical anodic charge that also matches the cathodic charge. Thus, contrary to some other applications in which Faradaic solvent reactions are detrimental to selective separations and should be suppressed^24^, it is shown here that for cathodic regeneration of PVF, water oxidation aids in the regulation of the solvent pH in favor of the equilibrium speciation of chromium and enhances the environmental remediation process.

### Mechanisms for selectivity towards HMOAs

**Fig. 3:**
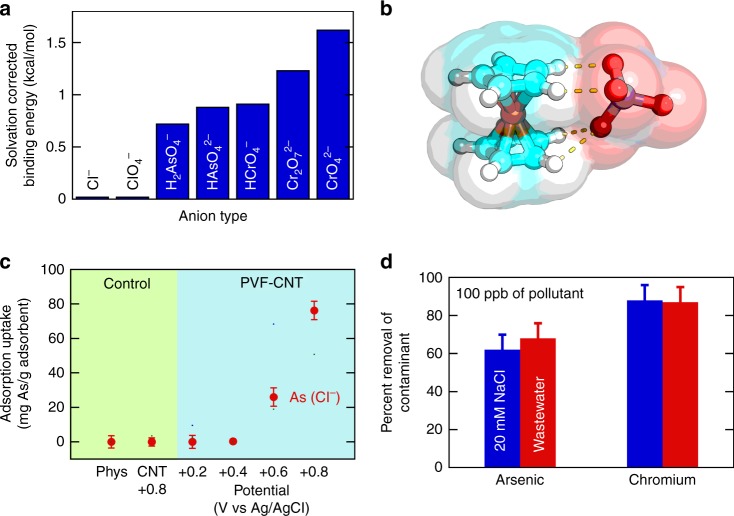
Mechanistic study of binding selectivity and generalization to arsenic contaminants. **a** Comparison of zero-temperature, solvation corrected binding energies calculated by DFT between ferrocenium and a range of oxyanions. The binding energy for chromium and arsenic-based anions is remarkably stronger than for the purely electrostatic attraction of chloride and perchlorate. **b** Electronic structure optimization of chromate (CrO_4_^2−^) with ferrocenium (Fc^+^), indicating that despite similar structural effects to perchlorate binding, the main governing contribution to selectivity is derived from charge transfer. **c** Adsorption capacity of arsenate at 1 mM KH_2_AsO_4_ in the presence of 20 mM NaCl, using PVF-CNT under different applied potentials. **d** Removal efficiency of chromium and arsenic under different water matrices, including a municipal secondary effluent solution spiked with Cr and As at 100 ppb for 120 min of charging (4 mg PVF on the electrode). Error bars represent standard deviations from three replicate measurements

Finally, the selectivity of the electrosorption step for HMOAs (chromium and arsenic) over chloride and perchlorate was investigated by density functional theory (DFT) calculations that allowed for water solvation effects. In Fig. 3a, we show the calculated binding energies between ferrocenium and a range of chromium and arsenic-containing oxyanions (Cl^–^, ClO_4_^–^, HCrO_4_^–^, CrO_4_^2–^, Cr_2_O_7_^2–^, H_2_AsO_4_^–^, and HAsO_4_^2–^). In contrast with previous DFT calculations for Fc under gas-phase conditions^23^, we explore here the strong screening effect of solvation for chloride or perchlorate, and the strong selectivity towards electron-rich HMOAs. The overall binding energy values in the present work are more accurate as these plane-wave-based calculations do not need to account for basis-set superposition errors under solvation^23^. For instance, the bindings between Fc^+^ and CrO_4_^2–^, Cr_2_O_7_^2–^, and HAsO_4_^2–^ are calculated to be 1.62, 1.22, and 0.91 kcal/mol, respectively, but only 0.018 and 0.015 kcal/mol for Cl^–^ and ClO_4_^–^, even though the geometry optimizations are similar for perchlorates, arsenates, and chromates (Fig. 3b). The complete geometry optimizations are shown in Supplementary Figure 19 and Supplementary Table 2. These results strongly imply an underlying electronic effect. The average O-H distance (Cl-H for Fc-Cl) for these structures also corresponds to the shorter *d*_O-H_ for cases with stronger binding (Supplementary Table 1).

A Bader’s charge analysis^32^ showed that a significant fraction of electrons were transferred from the HMOAs to the oxidized Fc centers (Supplementary Table 1). The strong binding of Fc^+^ with CrO_4_^2–^ and HAsO_4_^2–^ corresponded to more electron loss from the anions (0.30, and 0.41 |*e*|), indicating that charge transfer plays a dominant role in regulating binding selectivity. An atom-by-atom analysis of the electron transfer showed that the oxygens on the HMOAs are much more susceptible to losing electrons to ferrocenium than those from the non-HMOAs, for example, Cl^–^ or ClO_4_^–^, due to significant differences in their intrinsic electronegativities, as the ClO_4_^–^ (containing only halide and oxygen) is clearly more electronegative than CrO_4_^2–^ (containing a heavy metal center); thus, the electron cloud of ClO_4_^–^ is much less polarizable than that of CrO_4_^2–^, and much less attracted to Fc^+^. This electron cloud polarizability/charge transfer can be quantified in terms of the ionization potential of the solvated anions, IP = *E*_A_^0/–^ – *E*_A_^–/2–^, which can be interpreted as the energy required to remove one electron. A higher IP value indicates a higher barrier for electron transfer, roughly correlating with a weak Fc^+^ binding. Clearly, under water solvation, the calculated IP values of Cl^–^ and ClO_4_^–^ (>7.2 eV) are higher than those of the chromates and arsenates studied here. Furthermore, the IP values for CrO_4_^2–^ and HAsO_4_^2–^ are smaller than those for all other anions (5.6 and 5.2 eV), consistent with the higher charge transfer and stronger binding.

While qualitatively powerful, zero-temperature calculations cannot be compared directly with experimental binding energies. We performed entropy-corrected binding calculations to investigate the effect of finite temperatures on the intermolecular interactions and thereby provide more meaningful values for the binding energies. Owing to the heavy computational costs for the optimization of the structures for this task (see details in Methods), we selected Cl^−^, ClO_4_^–^, CrO_4_^2–^, and HAsO_4_^2–^ as validation cases to explore the importance of vibrational entropy in determining molecular selectivity. The binding energies of these four compounds with ferrocenium, accounting for both solvation and entropy corrections, are 2.90, 3.47, 5.53, and 4.73 kcal/mol, respectively. On comparison of these data with zero-temperature binding energies (0.02, 0.02, 1.62, and 0.91 kcal/mol, respectively), it is evident that vibrational entropy contributes greatly to the selectivity of the binding events. In addition, the overall trend in the interaction energies predicted by the zero-temperature calculations is preserved. These results further strengthen our mechanistic understanding of the electronic nature of the interactions, and of the strong charge transfer-mediated discrimination between these compounds. These guidelines are expected to provide important future design rules for HMOA capture, and establish redox electrodes as electrochemically efficient and molecularly tunable platforms for chemical and environmental separations.

Separation of arsenate indeed showed a significant selectivity and adsorption uptake in competition with chloride at 1 mM AsO_4_^−^, 20 mM NaCl (Fig. 3c), in agreement with theoretical calculations. XPS analysis indicated that arsenic, under +0.8 V adsorption and +0 V release remained in the pentavalent arsenate state (Supplementary Figure 15), while also confirming the strong selectivity over chloride by the surface elemental partitioning. The release experiments showed reversible performance equivalent to that of chromium (Supplementary Figure 12), with a desorption trend that supports the anionic bound state (higher release at more negative potentials). Finally, the removal efficiency for chromium and arsenic at 100 ppb was tested in the presence of a brackish concentration of sodium chloride (20 mM NaCl) and secondary effluent wastewater collected from the Deer Island Wastewater treatment plant (with a conductivity comparable to an ionic strength of ~20–30 mM). It was observed that >80% removal was achieved within 2 h of electrochemical adsorption (Fig. 3d), limited only by the capacity of the electrode.

### In situ electrochemical TEM studies

**Fig. 4:**
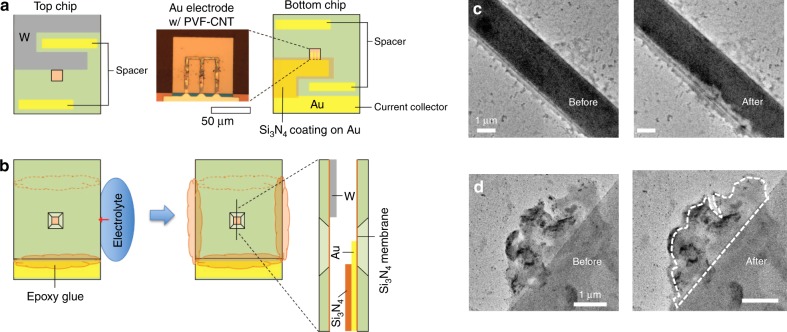
In situ TEM investigation of the redox-active metallopolymer under electrochemical modulation. **a** Schematic illustration of the top and bottom chips for the liquid cell. The optical micrograph image shows the PVF film deposited on the Au electrode patterned on the Si_3_N_4_ membrane window. **b** Chip assembly process. The two chips were overlapped and the two sides of the perimeter sealed with epoxy glue. The electrolyte was contacted with the side opening and injected into the cell via the capillary effect. A magnified cross-sectional view at the windows is illustrated. Detailed fabrication procedures for each component are given elsewhere^34^, with the polymer electrodeposition process described in Methods. **c** In situ TEM micrograph of PVF layer on top of gold chip. A solution of 20 mM NaClO_4_ was introduced and a CV @ 0.1 V/s was applied to the two-electrode system; a clear expansion of the PVF film can be seen following ion insertion (Supplementary Movie 1). **d** Swelling and contrast change of a PVF film under electrochemical modulation in the presence of 10 mM (NH_4_)_2_Cr_2_O_7_ (Supplementary Movie 3). See SI for all associated movies and cyclic voltammograms for Fig. 1c, d

Finally, the nanoscale behavior of our metallopolymer under electrical stimulus was studied through in situ TEM measurements in a liquid-confined cell (Fig. 4a, b). In situ electrochemical TEM has been introduced as a tool to study the kinetics and surface behavior of a variety of electrochemical materials and processes, ranging from lithium-ion batteries to electrodeposition^33–35^. From a fundamental perspective, investigations on the nanoscale behavior of metallopolymers under electrochemical control are of great importance for the understanding of adsorption mechanisms and future materials design. During electrochemical oxidation of the electrode materials, ion and solvent transfer can cause local swelling of the polymers, and affect electrochemical and chemical properties^36,37^. Indeed, studies of the behavior of thin-film Fc polymers under voltage and current response have played a central role in heterogeneous electrochemistry, as they provide a model system for fast electron transfer^38–40^ and dynamics of solvation^41–43^. PVF was electrodeposited from chloroform onto gold microelectrodes to form a thin uniform film (see Methods, Supplementary Information on microchip fabrication, and Supplementary Figures 1–6 for more details). Initially, 20 mM NaClO_4_ was introduced into the liquid cell as a model electrolyte for simulating brackish conditions. CV at a 0.01 V/s scan rate of up to 2 V revealed a sharp expansion of the polymer film once the oxidation potential of the Fc units was attained (Fig. 4c, with associated LSV in Supplementary Figure 7), with a 30% increase in thickness (see full kinetics of adsorption in Supplementary Movie 1). This expansion was attributed to the ingression of the counter anions together with their associated waters of solvation as Fc → Fc^+^, a phenomenon that has been inferred through indirect spectroscopic measurements^36^, but which is observed directly here through in situ microscopic imaging under TEM.

Desorption occurs on a much slower time-scale due to electron transfer and ion-pair dissociation kinetics during ion release and diffusional restructuring of the interfacial film as the ferrocenium (Fc^+^) reverts to Fc. Unlike purely organic polymers, metallopolymers were found to provide sufficient contrast for direct electron imaging due to the presence of the metal centers. Reversible ion uptake and desorption were clearly observed with dichromate (10 mM NH_4_Cr_2_O_7_) as well. Due to the higher contrast of the heavier Cr ion (Fig. 4d, Supplementary Movies 2–4), an up-concentration of the anion within the film could be seen upon application of an oxidative potential. Chromium binding was observed in a series of movies taken at different locations on the coated electrode (SI movies 1–3), indicating the fast kinetics of film swelling (<5 s). In the case of chromium, there is a shift in the oxidation potential of the PVF film towards lower potentials relative to those observed with the perchlorate anion, indicating the strong binding affinity of ferrocenium for chromate anions (Supplementary Figure 7). Finally, no second-phase particle growth was observed in the TEM measurements under the voltage ranges tested, indicating that molecular binding plays a major role, and that phase transformation processes are not significant under these conditions.

## Discussion

In summary, we have successfully investigated the anion-selective properties of the redox metallopolymer Fc for the removal of chromium and arsenic HMOAs from water under realistic operating conditions. PVF-CNT electrodes were used for the selective adsorption of two of the most prevalent and toxic HMOAs, with a high adsorption capacity (>100 mg/g at saturation) and remarkable regeneration properties. For chromium, a coupling of the release and regeneration of the electrodes results in transformation of the hexavalent pollutant into the less harmful trivalent form. From a practical perspective, this two-step electroswing process allows for the purification of a water stream under dilute pollutant concentrations while simultaneously reducing the byproduct toxicity. In addition, the system presents fast kinetics, and is an order of magnitude more active compared to various ion-exchange beds or even non-redox electrochemical systems, making it suitable for household and remote-site water purification, environmental remediation, and high-throughput industrial operations. In situ TEM measurements with an electrochemical liquid cell provided direct evidence of the redox-driven anion insertion into the poly(vinyl)ferrocenium film under charge, and the high affinity of the films for dichromate anions. Our work highlights the power of redox-mediated/Faradaic systems for the selective environmental capture and remediation of HMOAs, by showing strong binding selectivity and the possible coupling to an electron transfer process that mitigates the toxicity of the adsorbed element. In addition, the counter electrode reactions were found to be highly synergistic with the environmental transformation occurring at the metallopolymer electrode, pointing to the important role that electrochemical engineering can play in enabling an efficient reactive separation process. We also envision the use of these platforms for the recovery and valorization of waste streams in various chemical and electronic industries for a wider range of transition metal compounds.

## Methods

### PVF-CNT electrodes for electrochemical separation

The PVF-CNT electrodes were prepared following a drop-casting procedure^23,26^. A stock solution A of 80 mg PVF (Polysciences Inc.) and 40 mg multiwalled CNT (Sigma) was dissolved in 10 mL anhydrous chloroform, and a stock solution B of 40 mg of CNT in 10 mL chloroform was also prepared. The two stock solutions were sonicated for 2 h in icy water, and stocks A and B were mixed in a 1:1 ratio. Electrodes were drop cast using 50 μL of solution, and left to dry at 25 °C. For all systems, unless otherwise stated, the immersed electrochemical area was equivalent to 1 cm^2^ (1 cm by 1 cm) with 2 mg of PVF per electrode.

### Electrochemical adsorption

The electrochemical separations were carried out on a BASi cell in a three-electrode configuration with an aqueous Ag/AgCl reference electrode, and a Pt counter electrode, under ambient conditions. For adsorption and desorption tests, 5 mL of solution was used for the isotherm and equilibrium data measurements, and 8 mL for the kinetics tests. A background electrolyte concentration of 20 mM of either NaClO_4_ or NaCl was used. To quantify kinetics, 0.1 mL aliquots were taken at variable time points for ICP assay. All electrochemical studies were performed on a VersaSTAT 4 potentiostat (Princeton Applied Research) with automatic IR compensation between 5 Ω and 50 MΩ. The uptake values are reported as the adsorption capacity normalized by the mass of the polymer (2 mg of PVF per electrode, unless otherwise stated). For consistency, chronoamperometry was used for all the separation tests in this work, with chronopotentiometric measurements yielding comparable results.

### In situ TEM microchip

In situ TEM observation of the chromate adsorption/desorption on PVF-CNT film was performed on a liquid confining cell. The details of the cell dimensions and the fabrication procedure are provided elsewhere^34^. The liquid cell consisted of two separate chips with Si_3_N_4_ membrane windows (~70 nm thick). The top chip had a tungsten electrode (counter electrode) and the bottom chip a gold electrode (working electrode). The gold electrode was coated with Si_3_N_4_ except for the area on the membrane window to prevent the electrochemical reaction outside the field of view. The cells/microchips were then held by a mini-alligator clip at the gold current collector, and immersed into a 1 mg/mL PVF solution in chloroform, with 100 mM tetrabutylammonium hexafluorophosphate (TBAPF_6_^−^). Electrodeposition was achieved by the application of a +0.8 V vs. Ag/AgCl potential to the microchip for 5 min to achieve optimal coating. The coating was observed under a high-resolution optical microscope. The optimization of the process under different electrochemical conditions, electrolyte chemistries, and concentrations is given in the Supplementary Information (Supplementary Figures 1–6). Once the polymer film was deposited on the assembled chip, we injected the electrolyte and completely sealed the cell. The cell was mounted on a Nanofactory Scanning Tunneling Microscope–TEM holder. Autolab PGSTAT101 was connected to the holder to perform the electrochemical tests. We used JEOL 2010F (operated at 200 kV acceleration voltage) for the in situ TEM imaging. The configuration of the liquid cell chips and the assembly procedure are illustrated in Fig. 4a.

### Liquid-phase analysis

Inductively-coupled plasma optical emission spectroscopy: a PerkinElmer Optima 8000 ICP-OES spectrometer was used to quantify the concentrations of chromium and arsenic in solution. To establish a baseline for the ICP readings, a 2% w/w HNO_3_ solution was prepared from 70% HNO_3_ (Sigma-Aldrich, ACS reagent), and used as the solution into which the adsorbed species were electrochemically discharged, as well as to dilute any concentrated samples. Calibration solutions were made by diluting the ICP calibration standards (1000 ± 2 mg/L, 2% w/w HNO_3_) from Fluka Analytical, TraceCERT^®^ prepared 2% w/w HNO_3_ solution. A total of four calibration samples were prepared for each of the three cations examined, at concentrations of 10, 1, 0.5, and 0.1 mg/L (with 2% w/w HNO_3_ being 0 mg/L). After calibration, the linear fit was visually inspected, and the instrument was also set to perform a quality control check by resampling the 10 mg/L calibration solution to ascertain its calibrated concentration measurement. Each solution sample was measured in triplicate by the spectrometer to yield an averaged reading.

### Spectrophotometric analysis of Cr oxidation state

To measure the content of Cr(VI) in the aqueous samples, a one-step colorimetric assay was used (BioAssay Systems QuantiChrom Chromium Assay Kit, DCRM-250). The method relies on a mix-incubate-measure protocol, and the samples of the solution were pre-diluted to fit within the 0.02–2 mg/L range for the assay. Whereas the amount of Cr(VI) was directly measured, the amount of Cr(III) was estimated by the difference in the total amount of Cr from ICP relative to that of Cr(VI), and also confirmed by an acid treatment to convert all the Cr(VI) to Cr(III).

### Electronic structure calculations

We performed DFT calculations based on a generalized gradient approximation (GGA-PBE form) treatment of the exchange correlation method, implemented in the Vienna ab initio simulation package (VASP)^44,45^. The projector augmented-wave and plane-wave methods were used to describe core and valence electrons, respectively. The cutoff energy was set to be 400 eV, and in order to include the van der Waals interactions between moieties, we adopt Grimme’s DFT-D3 scheme^46^. Since the gas-phase calculations greatly overestimate the interaction between molecules, we applied an implicit water solvation model that describes the electrostatics, cavitation, and dispersion effects^47,48^. In order to obtain the ground state, we used multiple different geometric configurations as initial structures for each system, and then optimized them without any symmetry constraints. The binding energy is defined as BE = *E*_A_^–/2–^ + *E*_Fc_^+^ – *E*_AFc_^0/–^, where *E* refers to the total energy of the studied moiety. According to our calculations, the overall interaction trend in the gas phase and with solvation was the same, and as such, we base our discussions solely on the more physically relevant interactions with water solvation. In order to account for the entropy contribution to the binding between the ferrocenium and anionic moieties, we calculated their Helmholtz free energy at room temperature (300 K) with the solvation effect included. Following careful optimization of the structures with the convergence criteria of 1 × 10^–7^ eV and 1 × 10^–5^ eV/Å for the total energy and force components, respectively, the vibrational frequencies were calculated through perturbation of each atom in all three directions. The Helmholtz free energy can be written as:

$$F\left( T \right) = E\left( 0 \right) + \frac{1}{2}\mathop {\sum }\nolimits_s \hbar \omega _s + k_{\mathrm{B}}T\mathop {\sum }\nolimits_s {\mathrm{ln}}\left[ {1 - {\mathrm{exp}}\left( { - \hbar \omega _s/k_{\mathrm{B}}T} \right)} \right],$$

where the first term, *E*(0), represents the static energy at 0 K. The second and third terms correspond to the zero-point energy and vibrational energy at finite temperatures, respectively. The *ω*_*s*_ refers to the *s*th vibrational frequency. The room temperature binding energy BE (*T*), accounting for vibrational entropy, was then evaluated by a simple difference: BE(*T*) = *F*_Fc+_(*T*) + *F*_anion_(*T*) – *F*_Fc anion_(*T*). Variations in the temperature-dependent electron occupation and electron–phonon coupling were neglected. All these calculations were performed in VASP code.

## Electronic supplementary material

Supplementary Information

Description of Additional Supplementary Files

Supplementary Movie 1

Supplementary Movie 2

Supplementary Movie 3

Supplementary Movie 4

Supplementary Movie 5

## Data Availability

All relevant data are contained within the article, Supplementary Information and available from the authors upon reasonable request. The movies are uploaded as Supplementary Information to the paper, and have been compressed for reduced file size and ease visualization. Original files can be requested from the authors at x2su@mit.edu and kushima@mit.edu.
