# Supplementary material for: Electrochemically-mediated selective capture of heavy metal chromium and arsenic oxyanions from water
Source: Nat Commun. 2018 Nov 8;9:4701. doi: 10.1038/s41467-018-07159-0 (PMC6224381; doi:10.1038/s41467-018-07159-0)
Supplement: Supplementary file 1 — Supplementary Information [file 41467_2018_7159_MOESM1_ESM.doc]

Supplementary Information

**Electrochemically-mediated selective capture of heavy metal chromium and arsenic oxyanions from water**

Xiao Su^1^, Akihiro Kushima^2,3^, Cameron Halliday^1^, Jian Zhou^2^, Ju Li^2^, T. Alan Hatton^1*^

^1^Department of Chemical Engineering, MIT. 77 Massachusetts Ave, MA 02139, United States. ^2^Department of Nuclear Engineering, MIT. 77 Massachusetts Ave, MA 02139, United States.

^3^Present Address: Advanced Materials Processing and Analysis Center, Department of Materials Science and Engineering, University of Central Florida, 12760 Pegasus Drive, FL, 32816, United States

^*^E-mail: [tahatton@mit.edu](mailto:tahatton@mit.edu).

**Supplementary Methods**

**Materials Characterization.** A ZEISS Merlin High-Resolution SEM was used for the high resolution images in the main text, and the energy dispersive X-ray spectroscopy (EDS) was used to quantitate the amount of the different elements on the electrode. An accelerating voltage of 15-20 kV was used for the EDS measurements to ensure accurate capture of the iron and chromium peaks by EDS. The Physical Electronics Versaproble II X-ray Photoelectron spectrometer was used for the analysis of the surface of the electrodes.

**Micro-chip Preparation**. This sub-section shows all the different coating strategies for the micro-chip, and the strong effect of both polymer concentration, deposition time and supporting electrolyte chemistry.

**
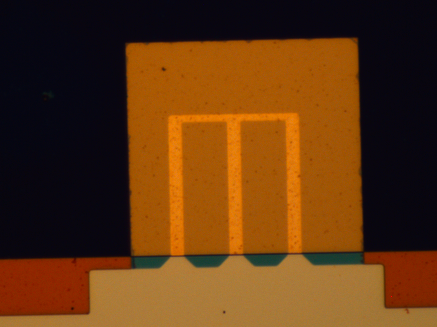
**

**Supplementary Figure 1.** Original Gold-Chip under Optical Microscope.


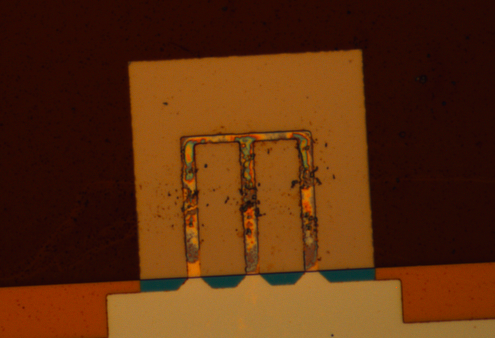


**Supplementary Figure 2.** Optimal electrodeposition using 0.4 mg/mL of 100 mM TBAPF_6_ for 5 min under +0.8 V.

**
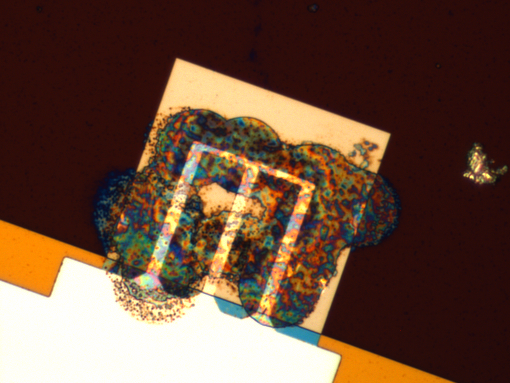
**

**Supplementary Figure 3**. +0.8 V vs Ag/AgCl, under 4 mg/mL of PVF in 100 mM TBAPF_6_ for 5 min.

**
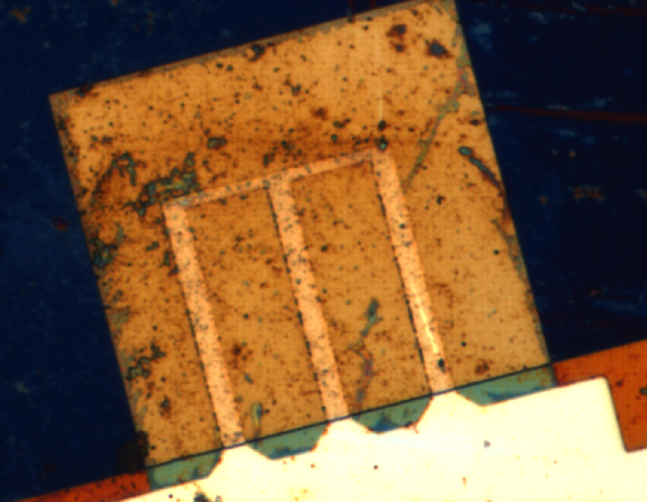
**

**Supplementary Figure 4**. +0.8 V vs Ag/AgCl, under 0.4 mg/mL of TBAPF_6_ for 30 s.

**
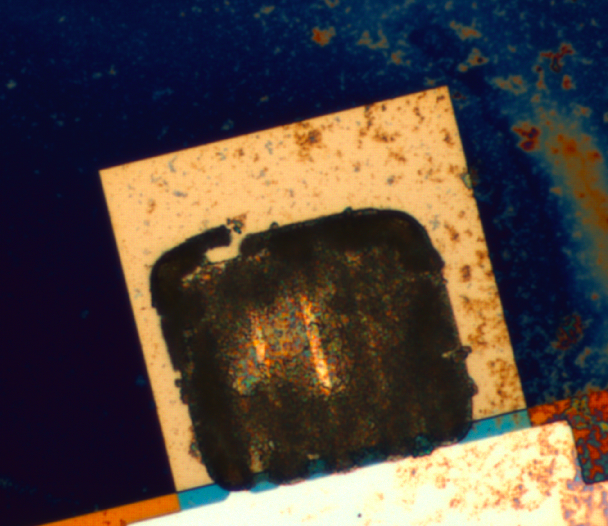
**

**Supplementary Figure 5**. +0.8 V vs Ag/AgCl, under 0.4 mg/mL of TBAClO_4_ for 5 min (chip used with 5-gold electrodes).


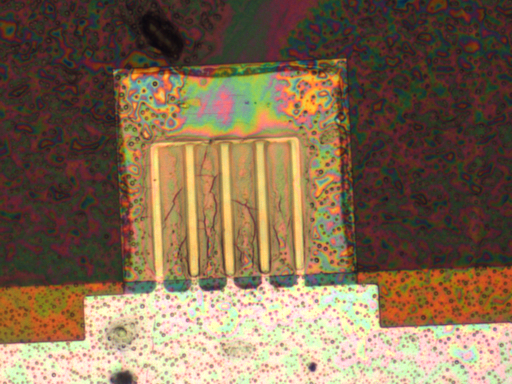


**Supplementary Figure 6**. Drop-cast of a 0.4 mg/mL solution of PVF, without electrochemical potential.

**Micro-chip In-situ Electrochemical Results.** This subsection presents the associated electrochemical data to the in-situ TEM results.

**Supplementary Figure 7**. Comparison of microchip response in the presence of 20 mM NaClO_4_ (left) and 10 mM NH_4_Cr_2_O_7_ in solution (right), under a 0.01 V/s scan-rate. Note the much higher charging current and also oxidation favorable shift of the oxidation peak. Bottom. Linear scan voltammetry (LSV) for the range in which the movies were taken.

**Electrochemical Measurements using PVF-CNT electrodes for Separations.**

**Supplementary Figure 8**. Charging of PVF-CNT electrode during adsorption in 1 mM (NH_4_)_2_Cr_2_O_7_, 20 mM NaClO_4_ at +0.8 V vs Ag/AgCl.

**Supplementary Figure 9**. Left. Discharge of PVF-CNT electrode, loaded with dichromate under conditions specified in Figure S8, at +0 V vs Ag/AgCl into clean electrolyte solution (20 mM NaClO_4_). Right. Charge passed through PVF-CNT at +0 V during regeneration.

**Supplementary Figure 10**. Adsorption kinetics of chromium by PVF-CNT, @ +0.8 V in the presence of 20 mM NaClO_4_ at different dichromate concentrations.

**Supplementary Figure 11**. Adsorption results of PVF-CNT working electrode at different electrode configurations, in 1 mM (NH_4_)_2_Cr_2_O_7_ in 20 mM NaCl, including a CNT counter and chronopotentiometric charging with a 2-electrode configuration.

**Supplementary Figure 12**. Arsenic Release Kinetics with PVF-CNT in the presence of 20 mM NaCl at different potentials, after adsorption @ +0.8 V in the presence of 1 mM KH_2_AsO_4_.

**Supplementary Figure 13**. XPS survey scan for (a) PVF-CNT after 10 mM dichromate adsorption, 20 mM NaClO_4_ supporting electrolyte, for 60 min at +0.8 V. (b) PVF-CNT after 1 mM dichromate adsorption, 20 mM NaClO_4_ supporting electrolyte. (c) CNT after 10 mM dichromate adsorption, 20 mM NaClO_4_ supporting electrolyte. (d) CNT after 1 mM dichromate adsorption, 20 mM NaClO_4_ supporting electrolyte.

**Supplementary Figure 14**. High resolution X-ray photoelectron spectroscopy analysis of the surface shows the presence of both Cr and Cl at the surface of PVF-CNT, while there is no Cr at the CNT surface, both experiments after 1 mM dichromate adsorption, 20 mM NaClO_4_ supporting electrolyte. In addition, the fully oxidized state of ferrocene can be noted from the Fe(III) peak for the PVF-CNT electrode.

**Supplementary Figure 15**.. High resolution X-ray photoelectron spectroscopy analysis of the surface of PVF-CNT after 1 mM HAsO_4_^2-^ adsorption in 20 mM NaCl, for 60 min charging at +0.8 V.

**Measurements of pH**

**Supplementary Figure 16**. Solution pH before, after adsorption and after release of chromium (for a swing of 30 min adsorption at +0.8 V vs Ag/AgCl, 30 min release at +0 V vs Ag/AgCl).

**Supplementary Figure 17.** Solution pH after electrosorption of the adsorption stock (in blue), at +0.8 V vs Ag/AgCl in 1 mM dichromate, 20 mM NaClO_4_ for 30 min. Solution pH of the release solution (20 mM NaClO_4_) for 30 min (in red). An increase in hydroxides can be seen during charging and an increase in protons can be seen during discharge. The error bars represent standard deviations from three replicates.

**Supplementary Figure 18.** Solution pH after electrochemical adsorption @ 1 mM dichromate, 20 mM NaClO_4_, for 60 min using PVF-CNT.

**Electronic Structure Calculation Results**

**Supplementary Table 1**. Summary of parameters from electronic structure calculations.

|  | Cl^–^ | ClO_4_^–^ | CrO_4_^2–^ | HCrO_4_^–^ | Cr_2_O_7_^2–^ | HAsO_4_^2–^ | H_2_AsO_4_^–^ |
| --- | --- | --- | --- | --- | --- | --- | --- |
| B.E. (kcal/mol) | 0.02 | 0.02 | 1.62 | 0.88 | 1.23 | 0.91 | 0.72 |
| *d*_O-H_ (Å) | 2.65 | 2.51 | 2.37 | 2.38 | 2.27 | 2.27 | 2.41 |
| Δ*Q* (|*e*|) | 0.10 | 0.02 | 0.30 | 0.03 | 0.15 | 0.42 | 0.06 |
| I.P. (eV) | 7.29 | 7.27 | 5.61 | 6.80 | 6.60 | 5.20 | 6.26 |

**Supplementary Figure 19**. Summary of optimized geometries (with solvation correction) between ferrocenium and the various anions.

**Supplementary Table 2**. Detailed xyz coordinates for optimized geometries.

Coordinates (*xyz*, in Å)

**Fc-Cl**

H 6.505 10.162 11.181

H 8.154 8.027 11.459

H 10.696 8.944 11.576

H 10.636 11.625 11.424

H 8.049 12.406 11.162

H 6.664 10.003 8.033

H 8.348 7.878 8.105

H 10.882 8.821 8.163

H 10.788 11.504 8.077

H 8.182 12.263 8.012

C 7.585 10.200 11.296

C 8.456 9.071 11.458

C 9.793 9.554 11.556

C 9.767 10.974 11.437

C 8.400 11.385 11.284

C 7.749 10.047 8.023

C 8.640 8.923 8.046

C 9.977 9.416 8.040

C 9.929 10.841 8.033

C 8.551 11.241 8.014

Fe 8.890 10.163 9.719

Cl 12.752 8.290 9.965

**Fc-ClO_4_**

H 6.100 9.594 11.130

H 7.755 7.445 11.375

H 10.281 8.382 11.680

H 10.200 11.057 11.645

H 7.633 11.834 11.298

H 6.418 9.689 7.968

H 7.916 7.422 8.127

H 10.515 8.157 8.292

H 10.646 10.832 8.237

H 8.127 11.809 8.041

C 7.174 9.625 11.292

C 8.047 8.490 11.430

C 9.377 8.982 11.620

C 9.334 10.403 11.600

C 7.981 10.810 11.394

C 7.502 9.639 8.024

C 8.294 8.441 8.095

C 9.668 8.825 8.158

C 9.737 10.245 8.133

C 8.405 10.758 8.053

Fe 8.544 9.626 9.779

O 13.087 9.394 8.407

O 14.427 9.167 10.377

O 12.414 10.451 10.441

O 12.308 8.068 10.235

Cl 13.061 9.271 9.866

**Fc-CrO_4_^–^**

H 5.913 9.651 11.263

H 7.480 7.455 11.362

H 10.080 8.287 11.397

H 10.104 10.964 11.337

H 7.506 11.834 11.258

H 6.002 9.702 7.981

H 7.467 7.434 8.048

H 10.092 8.146 8.144

H 10.233 10.832 8.103

H 7.693 11.809 7.984

C 6.998 9.648 11.343

C 7.826 8.485 11.386

C 9.189 8.915 11.395

C 9.202 10.354 11.355

C 7.841 10.802 11.325

C 7.087 9.646 7.957

C 7.862 8.446 8.003

C 9.242 8.815 8.054

C 9.317 10.253 8.036

C 7.981 10.761 7.974

Fe 8.283 9.614 9.684

O 12.015 9.404 9.326

O 14.481 9.383 10.457

O 12.447 10.776 11.641

O 12.415 8.040 11.650

Cr 12.841 9.400 10.780

**Fc-HCrO_4_**

H 5.582 9.676 10.978

H 7.163 7.517 11.459

H 9.668 8.424 11.991

H 9.645 11.086 11.871

H 7.129 11.901 11.233

H 6.229 9.649 7.867

H 7.714 7.399 8.220

H 10.279 8.158 8.645

H 10.403 10.823 8.563

H 7.906 11.785 8.084

H 13.119 8.967 8.770

C 6.634 9.699 11.251

C 7.467 8.558 11.517

C 8.779 9.033 11.829

C 8.767 10.454 11.754

C 7.449 10.875 11.397

C 7.302 9.609 8.035

C 8.089 8.419 8.208

C 9.445 8.815 8.413

C 9.509 10.236 8.374

C 8.189 10.738 8.143

Fe 8.154 9.643 9.890

O 12.593 9.608 9.288

O 14.260 9.112 11.485

O 12.020 10.578 11.806

O 11.862 7.914 11.424

Cr 12.691 9.285 11.062

**Fc-Cr_2_O_7_^–^**

H 5.948 9.716 11.531

H 7.518 7.504 11.368

H 10.098 8.344 11.058

H 10.121 10.997 11.019

H 7.556 11.894 11.310

H 5.507 9.646 8.393

H 7.095 7.451 8.171

H 9.650 8.321 7.753

H 9.639 10.974 7.709

H 7.081 11.842 8.106

C 7.030 9.705 11.429

C 7.862 8.535 11.350

C 9.217 8.969 11.196

C 9.230 10.395 11.179

C 7.883 10.857 11.319

C 6.575 9.646 8.190

C 7.415 8.484 8.066

C 8.751 8.934 7.822

C 8.745 10.360 7.798

C 7.407 10.808 8.029

Fe 8.007 9.668 9.638

O 13.739 9.398 9.519

O 13.876 9.401 6.748

O 11.905 10.792 7.930

O 11.840 8.105 7.927

O 12.285 8.132 11.551

O 14.582 9.378 12.158

O 12.408 10.816 11.530

Cr 12.810 9.426 7.991

Cr 13.235 9.432 11.235

**Fc-HAsO_4_^–^**

H 14.847 11.015 11.202

H 5.944 9.757 11.401

H 7.605 7.609 11.547

H 10.169 8.566 11.725

H 10.094 11.195 11.705

H 7.477 11.999 11.519

H 6.210 9.765 8.161

H 7.851 7.599 8.304

H 10.408 8.519 8.509

H 10.363 11.165 8.492

H 7.769 11.991 8.274

C 7.024 9.789 11.516

C 7.903 8.653 11.604

C 9.242 9.141 11.731

C 9.200 10.567 11.721

C 7.835 10.974 11.589

C 7.295 9.785 8.222

C 8.163 8.640 8.288

C 9.509 9.115 8.374

C 9.484 10.539 8.366

C 8.120 10.962 8.273

Fe 8.351 9.818 9.967

O 12.715 10.004 8.800

O 12.261 11.440 11.230

O 12.415 8.567 11.221

O 14.701 10.132 10.808

As 12.882 10.033 10.493

**Fc-H_2_AsO_4_**

H 13.615 9.136 8.570

H 14.554 11.054 11.327

H 5.692 9.752 11.387

H 7.359 7.605 11.567

H 9.898 8.571 11.888

H 9.781 11.226 11.944

H 7.196 11.990 11.648

H 6.126 9.774 8.139

H 7.750 7.602 8.409

H 10.291 8.515 8.736

H 10.239 11.192 8.650

H 7.684 11.989 8.271

C 6.763 9.784 11.563

C 7.645 8.649 11.657

C 8.975 9.147 11.866

C 8.911 10.571 11.909

C 7.556 10.967 11.720

C 7.206 9.789 8.249

C 8.064 8.642 8.393

C 9.410 9.120 8.535

C 9.384 10.543 8.471

C 8.032 10.960 8.300

Fe 8.167 9.819 10.063

O 13.241 10.023 8.753

O 11.817 11.467 10.628

O 12.029 8.535 10.846

O 14.212 10.140 11.409

As 12.666 10.035 10.472
