# Supplementary material for: Electrochemically-mediated selective capture of heavy metal chromium and arsenic oxyanions from water
Source: Nat Commun. 2018 Nov 8;9:4701. doi: 10.1038/s41467-018-07159-0 (PMC6224381; doi:10.1038/s41467-018-07159-0)
Supplement: Supplementary file 2 — Description of Additional Supplementary Files [file 41467_2018_7159_MOESM2_ESM.docx]

**Description of Additional Supplementary Files**

**File Name:** Supplementary movie 1

**Description:** PVF-CNT film at 10 mV/s with 20 mM ClO4.

**File Name:** Supplementary movie 2

**Description:** PVF-CNT film at 10 mV/s at 10 mM dichromate Location 1

**File Name:** Supplementary movie 3

**Description**. PVF-CNT film at 10 mV/s at 10 mM dichromate Location 2

**File Name**: Supplementary movie 4

**Description**: PVF-CNT film at 10 mV/s at 10 mM dichromate Location 3

**File Name**: Supplementary movie 5

**Description**: PVF-CNT film at 10 mV/s at 10 mM dichromate Location 4
